# Design and Fabrication of High-Frequency Piezoelectric Micromachined Ultrasonic Transducer Based on an AlN Thin Film

**DOI:** 10.3390/mi13081317

**Published:** 2022-08-14

**Authors:** Junbin Zang, Zheng Fan, Penglu Li, Xiaoya Duan, Chunsheng Wu, Danfeng Cui, Chenyang Xue

**Affiliations:** Key Laboratory of Instrumentation Science & Dynamic Measurement, Ministry of Education, North University of China, Taiyuan 030051, China

**Keywords:** MEMS fabrication, multiphysics coupling simulation, PMUT

## Abstract

A piezoelectric micromachined ultrasonic transducer (PMUT) is a microelectromechanical system (MEMS) device that can transmit and receive ultrasonic waves. Given its advantages of high-frequency ultrasound with good directionality and high resolution, PMUT can be used in application scenarios with low power supply, such as fingerprint recognition, nondestructive testing, and medical diagnosis. Here, a PMUT based on an aluminum nitride thin-film material is designed and fabricated. First, the eigenfrequencies of the PMUT are studied with multiphysics coupling simulation software, and the relationship between eigenfrequencies and vibration layer parameters is determined. The transmission performance of the PMUT is obtained via simulation. The PMUT device is fabricated in accordance with the designed simple MEMS processing process. The topography of the PMUT vibration layer is determined via scanning electron microscopy, and the resonant frequency of the PMUT device is 7.43 MHz. The electromechanical coupling coefficient is 2.21% via an LCR tester.

## 1. Introduction

Ultrasound [1] is a mechanical wave with a frequency greater than 20 kHz, and it must be propagated through a certain medium. Ultrasound exhibits the advantages of short wavelength and good directionality; thus, it can typically achieve range, nondestructive testing, medical imaging, and fingerprint recognition. Ultrasound is widely used in automobiles, consumer electronics, medical diagnosis and treatment [2], and industrial fields. A piezoelectric micromachined ultrasound transducer (PMUT) [3,4] is a microelectromechanical system (MEMS) [5] that uses the piezoelectric effect of piezoelectric films for electroacoustic conversion; it can realize ultrasonic transmission and reception of a certain frequency [6,7,8,9]. As sensors and actuators, ultrasonic transducers have different applications in the ultrasonic frequency range between 20 kHz and 40 MHz due to the different ultrasonic characteristics of various frequencies [10]. Ultrasonic waves with lower frequencies travel farther in the same medium, while ultrasonic waves with higher frequencies exhibit better performance in terms of resolution. These characteristics determine the application of ultrasonic waves in different frequency bands in various scenarios.

In the high-frequency range, PMUTs can be used in medical imaging [10,11,12], nondestructive testing, and fingerprint recognition [13,14,15]. Fingerprint-recognition technology is a crucial technology in the fields of consumer electronics and security. Ultrasonic fingerprint recognition exhibits many unique advantages over capacitive or optical fingerprint-recognition technologies, such as the ability of ultrasonic waves to scan through smartphone casings made of glass, aluminum, stainless steel, sapphire, or plastic. Moreover, ultrasonic can accurately identify fingerprints when dirt is present on the fingers. Ultrasonic fingerprint-recognition technology can identify the 3D details of fingerprints, such as fingerprint ridges and sweat pores. Given the high-resolution requirements of fingerprint-recognition technology, the operating frequency of ultrasonic fingerprint sensors is typically around 5 MHz to 15 MHz [16]. In consideration of the advantages of ultrasound in high-frequency operating bands, such as good directivity and strong directivity, PMUTs operating in such frequency bands can achieve higher imaging resolution [17,18,19,20]. They exhibit some advantages in fingerprint imaging, medical imaging and diagnosis, and many other applications. For PMUTs used in applications such as fingerprint imaging and medical imaging, researchers have recently conducted studies on the design and manufacture of PMUTs. Piezoelectric materials are a key part of piezoelectric transducers. Piezoelectric thin-film materials commonly used in PMUT preparation include PZT [13], AlN [16], ZnO [21,22], PVDF, etc. MEMS micromachining technology is required for the preparation of PMUT. First, piezoelectric thin-film materials should be compatible with CMOS and MEMS processes. Among many piezoelectric thin-film materials, AlN and ZnO can realize the compatibility of CMOS and MEMS microfabrication. Secondly, AlN has high strength at room temperature and is more stable at high temperature. Furthermore, compared with PZT materials, AlN does not contain the heavy metal lead, which makes it more environmentally friendly in industrial manufacturing. To sum up, compared with the existing piezoelectric thin-film materials, using aluminum nitride as the piezoelectric thin-film material of PMUT has great advantages.

In applications, such as fingerprint recognition, nondestructive testing, and medical imaging, as examples, high-frequency ultrasonic sensing must be sensed in a frequency band greater than 5 MHz to achieve the high resolution required in the application scenario. The realization of high-frequency ultrasonic sensing relies on high-frequency accurate ultrasonic emission. For the application requirements of small size, low energy supply, and high resolution, PMUTs fabricated via MEMS technology can achieve high consistency, small size, and low power consumption at high frequencies. Here, a high-frequency PMUT with a simple structure, simple process flow, and mass production is fabricated. Through the theoretical analysis of the PMUT and the use of multiphysics coupling simulation, the structure design and performance simulation of the PMUT are realized. For example, the influence of the structural parameters of the thin-film vibration layer on the PMUT’s characteristic frequency is studied through the parametric scanning of the parameters of the constructed PMUT model. By using a silicon-on-insulator (SOI) wafer as the basic structure of the PMUT, the MEMS process flow is designed and wafer fabrication is completed. The morphological characterization and impedance test of the PMUT are performed via scanning electron microscopy (SEM) and the use of an LCR tester, and the electromechanical coupling coefficient of the PMUT is calculated.

## 2. Modeling and Analysis

A PMUT realizes the conversion of electrical and acoustic signals through the piezoelectric effect of the piezoelectric film material. When a sound wave propagates in the medium, the sound wave acts on the thin-film part of a PMUT. Sound waves are mechanical waves; hence, these waves will cause the membrane to vibrate with the same frequency but different amplitudes. The piezoelectric film layer exhibits the characteristic of a positive piezoelectric effect; therefore, the piezoelectric film layer will generate electric charges. The upper and lower electrodes can collect the charge signals generated by the piezoelectric film layer. Conversion is completed from ultrasonic wave to electric charge signal. A PMUT can be regarded as the inverse of the aforementioned process in ultrasonic transmission mode. First, an electrical signal of a certain frequency is applied to both ends of the piezoelectric film material. The piezoelectric thin-film material deforms at the same frequency as the electrical signal due to converse piezoelectricity. The mechanical deformation of the film creates a pressure difference in the medium and conducts it outwards, generating sound waves. The ultrasonic transmission function of a PMUT is realized through the preceding process [3,23].

**Figure 1:**
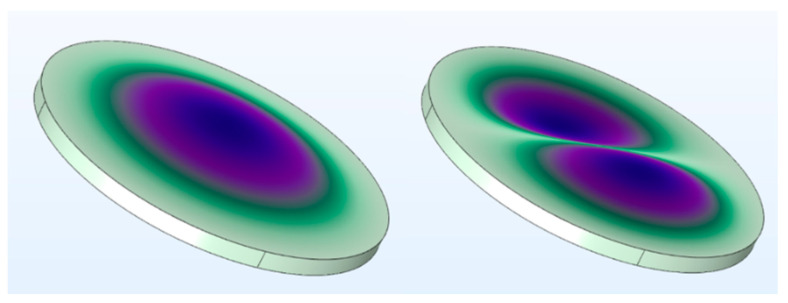
The first and second mode shapes of the thin plate.

Mechanical vibration is an intermediate process of electroacoustic conversion in the two working modes of a PMUT. The theoretical analysis of the mechanical vibration of a PMUT is crucial. Figure 1 shows the vibration modes of the piezoelectric thin plate. The piezoelectric thin plate is simulated and analyzed via finite element simulation, and the results indicate that the fundamental mode (0,1) exhibits better sound–vibration coupling efficiency than mode (1,1). On the basis of the force balance in the z-axis direction, the vibration of a circular thin plate with uniform thickness can be described as [24,25]

(1) $$D\nabla^{4}\left(w\left(r\right)e^{j\omega t}\right)+\rho \frac{\partial^{2}\left(w\left(r\right)e^{j\omega t}\right)}{\partial t^{2}}=0$$

where *w*(*r*) is the mechanical deformation amplitude of the thin plate at the radial distance *r* in the z-axis direction, *ω* is the operating frequency of the PMUT, *ρ* is the mass per unit area, and *D* is the bending stiffness of the plate. The bending stiffness *D* of the sheet can be expressed as [24]

(2) $$D=\int \frac{E\left(z\right)z^{2}}{1-v{\left(z\right)}^{2}}dz$$

where *E*(*z*) is the Young’s modulus of the sheet at *z* from the neutral axis, and *υ*(*z*) is the Poisson’s ratio of the sheet at *z* from the neutral axis. The neutral axis is the point where the sum of the stresses is zero. By integrating *z,* which is changing from the bottom to the top of the thin plate and setting the boundary of the circular thin plate to a completely fixed state, the resonant frequency of the first vibration mode can be obtained as [24]

(3) $${ƒ}_{1}=\frac{\sqrt{{\left(3.2/a\right)}^{4}\frac{D}{\rho }}}{2\pi }$$

where *a* is the radius of the circular sheet, and *ƒ*_1_ is proportional to the thickness and inversely proportional to the square of the radius [23].

(4) $${ƒ}_{1}\propto h/a^{2}$$

**Figure 2:**
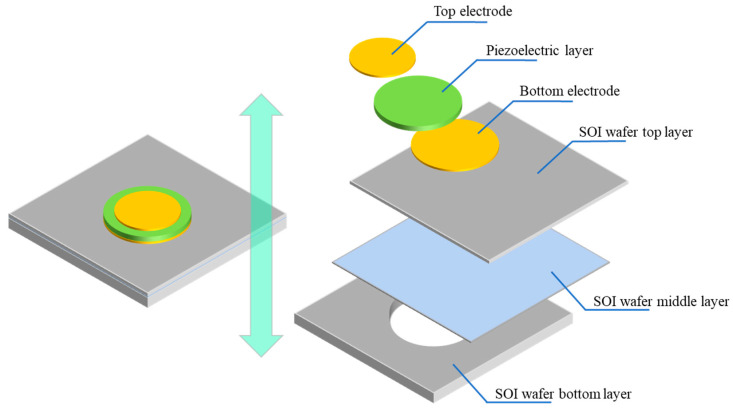
The structure of the array element of PMUT.

The basic structure of the PMUT is analyzed and designed after analyzing the resonant frequency. First, the aluminum nitride piezoelectric film is used as the piezoelectric film layer of the PMUT. The aluminum nitride film material can complete the ultrasonic receiving and transmitting functions of the PMUT through the positive and inverse piezoelectric effects, respectively. When the aluminum nitride piezoelectric film is deformed to generate charges or is excited by a high-frequency alternating electric field to drive the film to vibrate, collecting charges at both ends of the aluminum nitride piezoelectric film or generating a high-frequency alternating electric field is necessary to excite the film to vibrate. The top and bottom electrodes are designed on the upper and lower ends of the aluminum nitride piezoelectric film, respectively. The internal stress generated when the piezoelectric thin films are deposited onto thicker substrates with different lattice structures can cause the films to wrinkle, crack, and even peel off. Therefore, the aluminum nitride piezoelectric film typically has a certain thickness limit during the deposition process [26], making increasing PMUT resonance frequency with reference to Formula (4) difficult. In accordance with the neutral-surface principle, the aluminum nitride piezoelectric thin-film layer will generate lower stress and strain during vibration when the neutral surface of the thin-film vibration layer is in the aluminum nitride piezoelectric thin-film layer. This phenomenon will result in a lower charge output of the PMUT in ultrasonic receiving mode, reducing the receiving sensitivity of the PMUT. Therefore, the limitation of resonant frequency and the principle of the neutral layer can be combined to ensure the resonant frequency of the PMUT and improve its receiving sensitivity by adding a device support layer with a certain thickness at the lower end of the bottom electrode. The fabrication of the PMUT should be on a silicon substrate compatible with the MEMS microfabrication process to reduce processing difficulty and improve process compatibility. To ensure the consistency and controllability of back-cavity etching, designing an etch-stop layer at the bottom of the device-support layer, such as a silicon dioxide buried layer, is necessary. Therefore, the substrate of the PMUT is designed to be a SOI wafer. This wafer structure can meet many of the requirements of PMUTs that use MEMS microfabrication technology. Figure 2 shows the structure of a PMUT [27].

**Figure 3:**
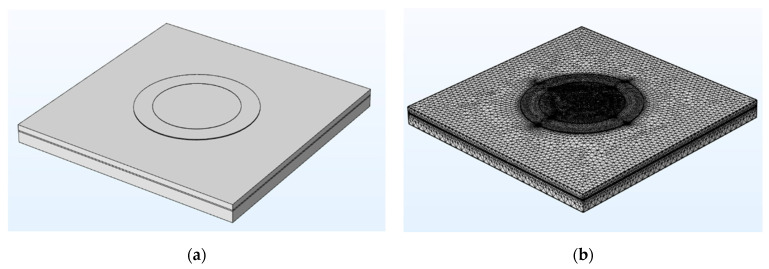
(**a**) PMUT array element structure geometric model; (**b**) meshing of PMUT geometric models.

The PMUT structure was simulated using COMSOL Multiphysics 5.5. First, the PMUT is geometrically modeled in accordance with the PMUT structure shown in Figure 2. The geometric parameters of the PMUT model are provided in Table 1. Figure 3 presents the geometric model of the PMUT structure. The steps for establishing the geometric model of the PMUT structure are as follows. ① The SOI-support-layer part is built. Cuboid geometry is used to build a solid cuboid with the length and width of LOS and the height of SOIB. Cylinder geometry is used to build a solid cylinder with radius BCR and height SOIB. The solid cuboid is subtracted from the solid cylinder via the Boolean operation to complete the construction of the SOI-support layer. ② The SOI buried-oxide-layer part is built. The cuboid geometric model is used to build a solid cuboid with the length and width of LOS and the height of SOIM to complete the SOI buried-oxide layer. ③ The SOI-device-layer part is built. The cuboid geometric model is used to build a solid cuboid with the length and width of LOS and the height of SOIT to complete the SOI-device-layer part. ④ The bottom electrode part is built. The construction of the bottom electrode is completed by establishing a solid cylinder with the radius of BER and the height of BET by using the cylindrical geometric model. ⑤ The piezoelectric film-layer part is built. A solid cylinder with the radius of PLR and the height of PLT is constructed using the cylindrical geometric model to complete the construction of the piezoelectric thin-film layer. ⑥ The top electrode section is built. A solid cylinder with the radius of TER and the height of TET is constructed using the cylindrical geometric model to complete the construction of the top electrode [28,29].

In accordance with the design of the basic structure of the PMUT device, the molybdenum metal, aluminum nitride, silicon, and silicon dioxide required by the geometric model of the PMUT array element are selected from the library of materials. The SOI-device and -support layers are made of silicon. The SOI-buried-oxygen layer is made of silicon dioxide. The piezoelectric film layer is made of aluminum nitride. The top and bottom electrodes are made of molybdenum metal.

After completing the setting of the material, the physical field structure distribution of the PMUT, the boundary conditions, and the meshing, the PMUT characteristic frequency is simulated.

## 3. Results and Discussion

### 3.1. Simulation Results

**Figure 4:**
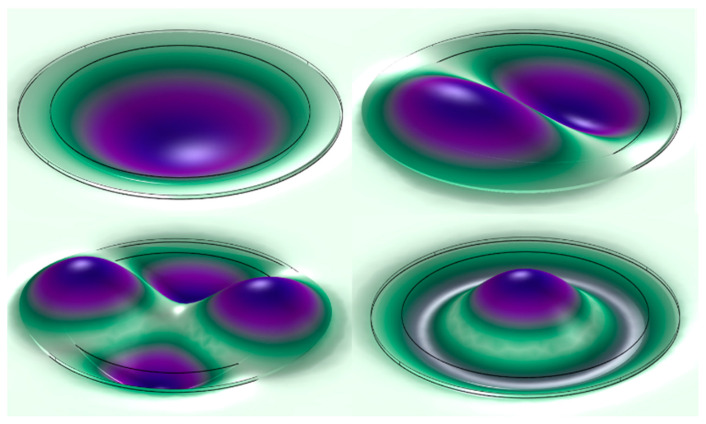
Mode shapes of the first four modes of PMUT.

In PMUTs, the characteristic frequency is the resonant frequency of the thin-film layer. The geometric model of the PMUT array element is simulated and calculated with multi-physics simulation software. The table and figure show the eigenfrequency values of the PMUT geometric model and the corresponding mode shapes. As shown in Figure 4, when the thin-film vibration layer of the PMUT array element geometric model is circular, the mode shapes of the PMUT geometric model are presented, which are the first-order, second-order, third-order, and fourth-order vibration modes. The resonant frequency of the first-order vibration mode is 7.27 MHz, and the mode shape is the thin-film vibration layer of the PMUT array element to generate an overall tent-like vibration up and down along the z-axis. The resonant frequency of the second-order vibration mode is 14.71 MHz, and the mode shape is the diameter of the PMUT as the boundary. Both ends of the boundary produce tent-like vibrations up and down along the z-axis. The resonant frequency of the third-order vibration mode is 24.00 MHz, and the mode shape is defined by the two perpendicular diameters of the thin-film vibration layer of the PMUT array element as the boundary. The four parts generate tent-like vibrations up and down along the z-axis. The resonant frequency of the fourth-order vibration mode is 27.45 MHz. The center and edge of the thin-film vibration layer whose mode shape is the PMUT array element vibrate up and down along the z-axis. The center is tent-shaped, and the edge is annular.

**Figure 5:**
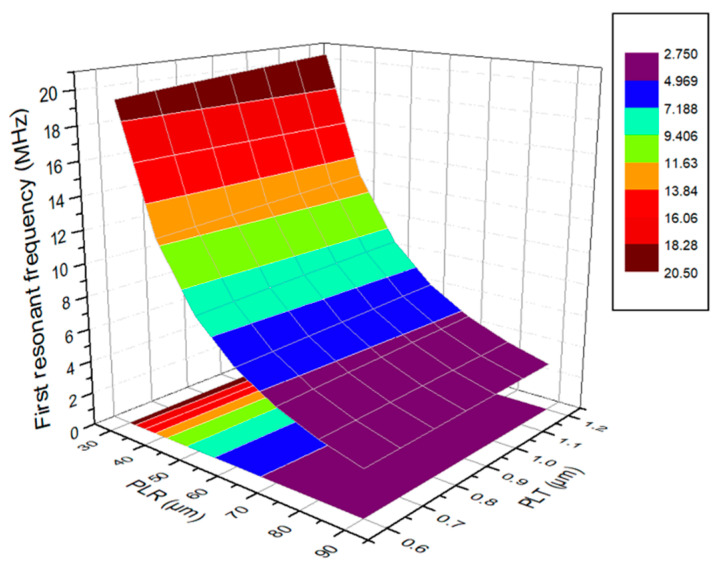
PMUT frequency vs. parameter plot.

The thin-film vibration layer is circular, and the first-order resonant frequency that uses the PMUT geometric model in Table 1 is 7.27 MHz. The PMUT array element structure can be optimally designed via the parametric scanning of the preset parameters. Parametric scanning is performed on the PLR and PLT parameters, in which the thin-film vibration layer is a circle. The values of the parametric scanning are provided in Table 2. Figure 5 illustrates the relationship between the first-order resonant frequency of the PMUT array element structure and a circular thin-film vibration layer and the relationship between PLR and PLT. The circular first-order resonant frequency evidently decreases with an increase in PLR. As PLT increases, the first-order resonant frequency increases slightly, because a thicker SOI-device layer is present in the PMUT array element structure as a device-support layer. Therefore, thickness changes that are less than 1 μm exert minimal effect on resonant frequency.

**Figure 6:**
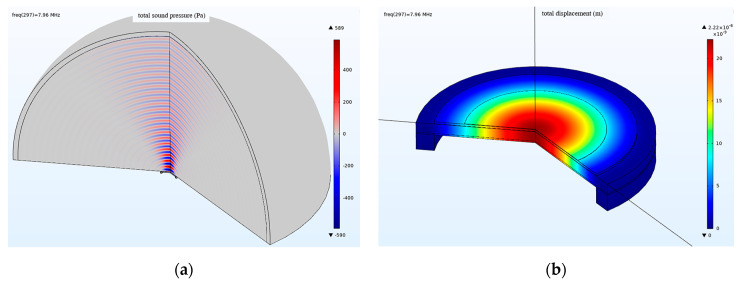
Simulation results of sound-emission performance of PMUT: (**a**) sound-pressure contours of the circular PMUT; (**b**) displacement contours of the circular PMUT in transmit mode.

Through frequency domain studies, calculations and simulations are performed for the frequencies of 5–12 MHz. The ultrasonic emission performance of the circular PMUT device with a thin-film vibration layer is studied, including the maximum sound pressure and displacement. The influence of the two PMUT device array elements on the ultrasonic emission direction under the condition of different spacings is also investigated. Figure 6a shows the 3D nephogram of the maximum sound pressure of the PMUT device in ultrasonic emission working mode at 5–12 MHz. The maximum sound pressure is 589 Pa, within the frequency range of 5–12 MHz and under an excitation of 1 V. The frequency of the maximum sound pressure appears at 7.96 MHz. Figure 6b shows the 3D cloud diagram of the displacement when the thin-film vibration layer of the PMUT device is circular. The displacement is the largest in the ultrasonic transmission mode at 5–12 MHz. The maximum displacement is 2.22 × 10^−8^ m within the frequency range of 5–12 MHz and under a voltage excitation of 1 V. The frequency of maximum displacement occurs at 7.96 MHz.

### 3.2. PMUT Fabrication

**Figure 7:**
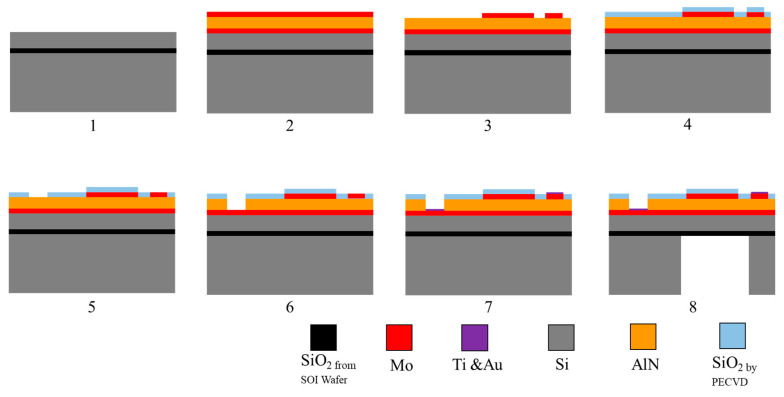
PMUT micromachining process flow.

In accordance with the basic structure design of the PMUT device described in Section 2, a customized SOI wafer is selected as the substrate of the PMUT device. The thickness of the customized SOI-wafer device layer is 5 μm; the thickness of the buried oxide layer is 1 μm; the thickness of the support layer is 475 μm. In addition, on the backside of the SOI wafer, under the SOI-support layer, there is a silicon dioxide layer with a thickness of 1 μm for balancing the stress of the SOI wafer. The total thickness of the SOI wafer is 482 microns, and the error of each part does not exceed 0.1 microns. The MEMS micromachining process is performed on basis of the SOI wafer [27]. The proposed process flow is illustrated in Figure 7.

① The SOI wafer is cleaned. The growth material has high requirements for the cleanliness of the wafer surface, and thus, the wafer should be cleaned. In this tapeout, acetone, anhydrous ethanol, and deionized water were used for the 5 min of ultrasonic cleaning to remove organic substances on the surface of the SOI wafer.

② The bottom electrode, piezoelectric film layer, and top electrode are sputtered onto the surface of the SOI wafer. First, aluminum nitride is deposited on top of the SOI-device layer. The aluminum nitride seed layer has a regular lattice structure and minimal defects, and it can effectively improve lattice mismatch between the piezoelectric thin-film layer and the substrate through the regular arrangement of the lattice of the seed layer, reducing internal stress and thermal mismatch. Simultaneously, the piezoelectric properties of the aluminum nitride piezoelectric thin-film layer and the adhesive force of the substrate can be improved. The materials are sequentially sputtered onto the SOI-device layer by using a special aluminum target and magnetron sputtering in a gas environment: 0.2 μm bottom electrode molybdenum metal, 1.2 μm piezoelectric film layer aluminum nitride, and 0.2 μm top electrode molybdenum metal.

**Figure 8:**
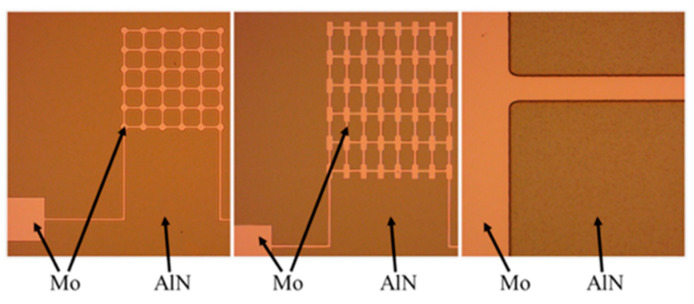
Optical microscope images of wafers completed with IBE.

③ The top electrode is patterned. The photolithography development area is large, and thus, more than 70% of the wafer surface photoresist must react with the development, and a relatively long development time is required. In this development and microscopic inspection, paying attention to whether the photoresist at the mask must be directly peeled off is necessary. When the microscopic examination and development effect are good, the wafer is post-baked, and the solvent in the photoresist on the wafer surface will completely evaporate after post-baking, better masking the etching. The post-baked wafer is etched via ion beam etching (IBE), and the etching time is 18 min. By using a resistance meter to measure the area after etching, the area is determined to be nonconductive. By using a metallographic microscope to observe the surface of the wafer, the top electrode molybdenum and aluminum nitride exhibit a clear boundary, and the minimum structure of the top electrode in the wafer does not appear to fall off and break. Figure 8 shows an optical microscope photograph of the wafer with completed IBE.

④ A protective layer of silicon dioxide is deposited. After cleaning and drying the wafer, ensuring that its surface is clean and free of contamination, plasma-enhanced chemical vapor deposition equipment is used to deposit 0.2 μm of silicon dioxide onto the surface of the wafer. Its function is to protect the exposed aluminum nitride on the wafer surface from being etched by the alkaline developer, and it acts as a mask for wet-etching in the next step. A resistance test is performed on the upper-electrode test site by using a resistance meter. The test results show no conduction, proving that the deposition of silicon dioxide onto the surface of the wafer is completed.

**Figure 9:**
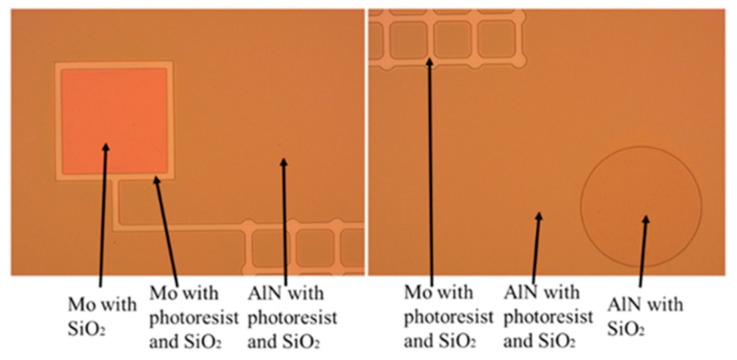
Optical microscope images of wafer surface photoresist at electrode pads.

⑤ The top and bottom electrode pads are etched. After silicon dioxide is deposited, it must be etched onto the lower electrode pads. The purpose of this procedure is to expose the lower electrode pad on the wafer surface through the subsequent etching, ensuring the lead of the lower electrode pad. After development, microscopic examination is performed. Figure 9 shows an optical microscope image of the wafer surface photoresist at the top and bottom electrode pads. The top and bottom electrode pads are etched for 12 min via reactive ion etching (RIE) to complete the etching of the silicon dioxide protective layer. Through microscope inspection and resistance test, the mask on the wafer surface is determined to be not conductive, but it is conductive in the upper electrode pad test, proving that RIE is completed.

⑥ The bottom electrode pad is etched. Silicon dioxide is used as a mask; AZ400K is used as an etching solution, and a dilute aqueous solution of AZ400K:H_2_O = 1:4 is used to etch aluminum nitride at 65 °C.

⑦ The pads of the top and bottom electrodes are sputtered with metals. After the wet-etching of aluminum nitride, the gold required for gold wire bonding should be sputtered onto the upper and lower electrode pads exposed on the wafer surface. Gold deposition onto the electrode pads is achieved using a lift-off method. First, a photolithography process is performed on the wafer surface, and then the wafer surface is shielded by steps, such as homogenization, exposure, and development. Metal sputtering is directly performed on the developed and blow-dried wafer, and 20 nm of titanium and 200 nm of gold are sputtered. After sputtering is completed, the wafer with the intact metal film on the surface is placed in acetone, and the photoresist on the surface is dissolved. The metal thin film remains on the wafer surface without being blocked by the photoresist, and the metal sputtering of the electrode pad is completed.

⑧ Back-cavity etching film release. After the sputter lift-off process, the wafer’s front-side process is completed. The front and back of the wafer are cleaned and blow-dried. The SOI wafer is deposited with 1 µm of silicon dioxide on the back to balance SOI-wafer stress. Therefore, the back-sealed SOI is first etched through the photoresist mask via RIE to expose the support-layer silicon that should be etched. After etching the SOI back-sealing silicon dioxide, the wafer is fully cleaned, and the residual photoresist is cleaned and dried. Back-cavity etching requires about 470 µm, and thus, the verticality of the etched sidewalls is extremely high. Choosing a shorter deep silicon etch cycle can increase the verticality of the sidewall and ensure the complete release of the film in accordance with design size. After homogenizing at low rotational speed, the AZ4620 photoresist is measured as 9.8 μm thick. The etching selectivity ratio of silicon and the photoresist in the deep silicon etching process is greater than 100:1; thus, the etching-selectivity ratio of silicon and silicon dioxide is greater than 100:1. Accordingly, a 9.8 μm photoresist and a 1 μm silicon dioxide secondary mask are sufficient to resist the whole process of deep silicon etching. By using a silicon wafer to perform the deep silicon etching process, 10 Bosch process cycles are performed. The stepper test shows that the etching height of different surface shapes is 20 μm, and the etching height of silicon is 2 μm in each cycle. Therefore, after 20 cycles of engraving, i.e., 265 cycles, the height information shows that the verticality of the steps is good, and no residual silicon is found on the bottom surface. The resonant frequency consistency between the elements provides a guarantee for improving the transmission and reception performance of the PMUT.

### 3.3. PMUT Morphology and Test

**Figure 10:**
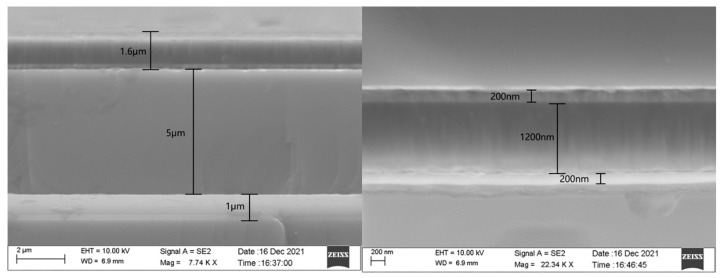
SEM image of a thin-film vibration layer and PMUT piezoelectric thin-film layers of PMUT.

SEM is conducted to observe the PMUT section, and the field-emission electron microscope used is a ZEISS SUPRA-55 with a resolution of 0.8 nm @ 15 kV. Figure 10 shows an SEM image of the SOI buried oxide layer and the SOI device layer [30,31]. The thickness of the SOI-buried-oxide layer is clearly 1 μm, while that of the SOI-device layer is clearly 5 μm. The thin-film vibration layer includes a bottom electrode, a top electrode, and a piezoelectric thin-film layer, with a total thickness of 1.6 μm. Figure 10 shows an enlarged SEM image of the thin-film vibration layer of the cross section of the PMUT device, which includes a top electrode, a piezoelectric thin-film layer, and a bottom electrode. As observed from the SEM image, the thickness of the top electrode is 200 nm, that of the piezoelectric thin-film layer is 1.2 μm, and that of the bottom electrode is 200 nm.

To characterize the performance of the PMUT, an impedance test is performed on the PMUT by using an LCR tester [32]. LCR tests are performed on PMUTs with a circular thin-film vibration layer and a radius of 55 μm.

**Figure 11:**
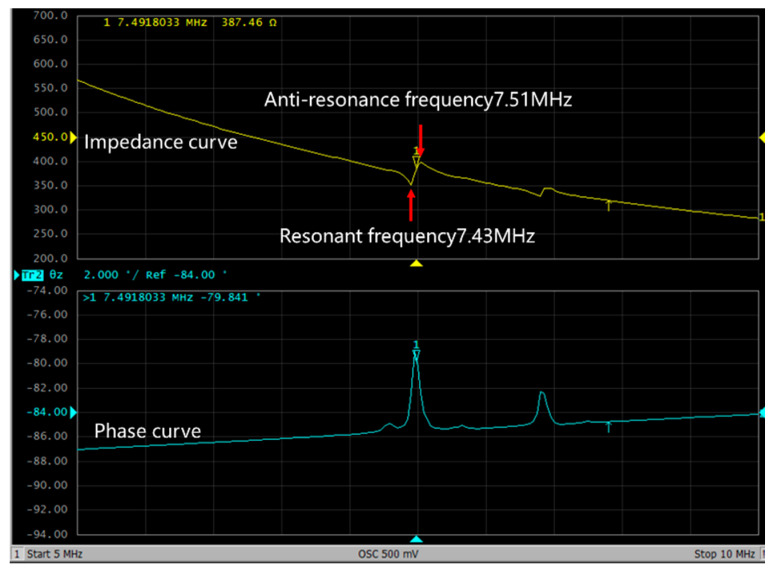
Impedance curve and phase curve of PMUT.

Figure 11 shows the impedance and phase curves of the PMUT. The resonant frequency of the first-order vibration mode of the PMUT device whose thin-film vibration layer is circular and whose radius is 55 μm is 7.43 MHz due to the limited verticality of the sidewalls during the release step of the deep silicon etched film, as shown in Figure 11. After the film is released, the radius of the thin-film vibration layer of the PMUT device is increased to a certain extent compared with the radius of the thin-film vibration layer in the design of the PMUT device. In accordance with the influence of the radius parameter on the resonant frequency of the PMUT device in Figure 5, the increase in the radius of the thin-film vibration layer of the PMUT device leads to a certain decrease in the resonant frequency of the PMUT device. Therefore, by comparing the simulation results of the resonant frequency with the actual test results, the actual test results of the resonant frequency are reduced to a certain extent compared with the simulation results.

As shown in Figure 11, the resonant frequency and anti-resonant frequency of the PMUT device with a circular thin-film vibration layer and a radius of 55 μm are 7.43 MHz and 7.51 MHz, respectively. The electromechanical coupling coefficient of the PMUT can be calculated from the impedance curve. The formula for calculating the electromechanical coupling coefficient of PMUT is [32]

(5) $$K_{eff}^{2}=1-{\left(\frac{f_{r}}{f_{a}}\right)}^{2}$$

where *ƒ_a_* is the anti-resonant frequency of the PMUT device, and *ƒ_r_* is the resonant frequency of the PMUT device. In accordance with the preceding formula, the electromechanical coupling coefficient of the PMUT device with a circular thin-film vibration layer and a radius of 55 μm can be calculated as 2.12%.

PMUT resolution refers to the ability of the PMUT sensor to distinguish between two adjacent interfaces, which are divided into vertical resolution and horizontal resolution. The vertical resolution refers to the minimum depth distance between the front and rear points on the axis of the sound beam. The vertical resolution is related to the ultrasonic wavelength, and in the same medium, the higher the frequency of the ultrasonic wave, the shorter the wavelength. This results in a smaller vertical resolution of the ultrasonic sensor. According to the formula [33,34]

(6) $$\lambda =\frac{V}{f}$$

*λ* is the wavelength of the ultrasonic wave, *ƒ* is the frequency of the ultrasonic wave, and *V* is the propagation speed of the ultrasonic wave in the medium. When the high-frequency PMUT works in the air, the ultrasonic wave speed *V* is 340 m/s, and, when the center operating frequency *ƒ* of the PMUT is 7.5 MHz, the wavelength is 45.33 microns. In the ultrasonic propagation process, due to the existence of diffraction, the wavelength must be smaller than the size of the detection target to form reflection. That is to say, it must be greater than 1/2 of the wavelength to produce reflection. The smallest size that PMUT can recognize is 22.665 microns.

Due to the characteristics of the fast attenuation of high-frequency ultrasonic waves, the effective measurement distance will be shorter than that of low-frequency ultrasonic waves. According to the above characteristics, high-frequency PMUT can be used for distance measurement and imaging at short distances and requiring higher precision, such as precise flaw detection on small objects, interventional medical diagnosis, and the detection and imaging of small gaps and pipes.

## 4. Conclusions

In this work, a piezoelectric micromachined ultrasonic transducer based on an AlN thin-film material was designed using multiphysics simulation software. The influence of the parameters of the thin-film vibration layer on the characteristic frequency is studied. The designed PMUT first-order resonance frequency reaches 7 MHz. The SOI wafer is used as the basic structure of PMUT, and the PMUT is fabricated by the MEMS processing process. The first-order resonance frequency of the fabricated PMUT reaches 7.43 MHz, and the electromechanical coupling coefficient reaches 2.12%. In future work, we will focus on PMUT emission sound pressure testing and different high-frequency sensing applications.

## Figures and Tables

**Table 1:** The geometric parameters of the PMUT model.

Parameters Name	Numerical Value (μm)	Description
TER	38	Top electrode radius
TET	0.2	Top electrode thickness
BER	55	Bottom electrode radius
BET	0.2	Bottom electrode thickness
PLR	55	Piezoelectric layer radius
PLT	1.2	Piezoelectric layer thickness
SOIT	5	SOI-wafer top-layer thickness
SOIM	1	SOI-wafer middle-layer thickness
SOIB	10	SOI-wafer bottom-layer thickness
BCR	55	Back-cavity radius

**Table 2:** Parametric sweep value.

Parameter Name	Parametric Sweep Value (μm)
PLR	30, 35, 40, 45, 50, 55, 60, 65, 70, 75, 80, 85, 90
PLT	0.6, 0.7, 0.8, 0.9, 1.0, 1.1, 1.2

## Data Availability

The data presented in this study are available on request from the corresponding author.
